# Advantages of Mohs Surgery in the Treatment of NMSC in the Head and Neck District

**DOI:** 10.3390/jcm14134732

**Published:** 2025-07-04

**Authors:** Valentina Celoria, Francois Rosset, Ginevra Pertusi, Simone Ribero, Pietro Quaglino, Massimo Gattoni, Rossana Tiberio

**Affiliations:** 1 Dermatologic Clinic, Department of Medical Sciences, University of Turin, 10124 Turin, Italypietro.quaglino@unito.it (P.Q.); 2 Dermatology C.S., Sant’ Andrea Hospital, ASL Vercelli, 13100 Vercelli, Italy

**Keywords:** Mohs surgery, aesthetic results, long-term cost effectiveness, oncologic outcomes, standard excision

## Abstract

This narrative review examines the efficacy, cost-effectiveness, and aesthetic outcomes of Mohs micrographic surgery (MMS) compared to standard excision for treating non-melanoma skin cancers (NMSCs). A comprehensive literature search was conducted across multiple databases, including PubMed, Scopus, and Cochrane Library, covering studies published from 2000 to 2024. Key terms such as “Mohs Micrographic Surgery,” “non-melanoma skin cancer,” “recurrence rates,” “cost-effectiveness,” and “aesthetic outcomes” were utilized. Inclusion criteria encompassed peer-reviewed articles, clinical trials, and observational studies focusing on MMS and standard excision outcomes. Exclusion criteria included studies with inadequate data or those not published in English. The review highlights the superior oncologic outcomes of MMS, its cost-effectiveness over the long term, and comparable aesthetic results to standard excision principally. **Methods**: This narrative review was conducted following established guidelines for reporting narrative reviews. A systematic search strategy was employed across selected databases, with the last search conducted in May 2025. The search terms used were “Mohs Micrographic Surgery,” “non-melanoma skin cancer,” “recurrence rates,” “cost-effectiveness,” and “aesthetic outcomes.” Studies included were published between 2000 and 2024, in English, and provided data on the specified outcomes. **Results**: The majority of studies indicated that MMS offers superior recurrence-free survival rates compared to standard excision. Regarding cost-effectiveness, MMS was found to be more economical over the long term due to reduced recurrence rates and the need for fewer re-excisions. Aesthetic outcomes were comparable between MMS and standard excision, with both methods yielding satisfactory results. **Discussion**: The findings of this review support the use of MMS as a preferred treatment for high-risk NMSCs, particularly in cosmetically sensitive areas. While MMS may involve higher initial costs, its long-term cost-effectiveness and superior oncologic outcomes justify its use. The aesthetic outcomes associated with MMS are comparable to those of standard excision, making it a viable option for patients concerned with cosmetic results. **Limitations**: This review acknowledges several limitations, including the heterogeneity of study designs and potential selection biases inherent in the included studies. Additionally, the absence of randomized controlled trials comparing MMS and standard excision directly limits the strength of the conclusions drawn. **Conclusions**: This narrative review underscores the advantages of MMS in treating high-risk NMSCs, particularly in terms of recurrence rates and long-term cost-effectiveness. While both MMS and standard excision offer comparable aesthetic outcomes, the superior oncologic results of MMS make it a preferable option in certain clinical scenarios.

## 1. Introduction

Skin cancer is the most commonly diagnosed type of cancer, of which basal cell carcinoma (BCC) is the most common. Surgery is typically the first-line treatment for non-melanoma skin cancer (NMSC).

The impact of facial skin cancer surgery on patients’ perception of their aesthetic appearance remains insufficiently understood. However, it may lead to extensive facial defects and visible scarring, which can negatively affect patients’ overall well-being. Treatment options for NMSC include standard surgical excision (SE) and Mohs micrographic surgery (MMS) as we can see in Table 1 [1].

MMS is specifically designed to preserve healthy tissue by providing a detailed evaluation of the surgical margins [1].

While standard excision is a widely used approach, it may not always guarantee complete removal of the tumor, especially in cases with poorly defined borders. In contrast, Mohs micrographic surgery is a more precise method that involves the systematic removal and immediate microscopic examination of tissue layers, ensuring that all cancerous cells are excised while minimizing the removal of surrounding healthy tissue. This technique is particularly advantageous for tumors located in cosmetically sensitive areas, such as the face, as it enhances both the effectiveness of the treatment and the aesthetic outcome as we can see in Table 2 [2].

## 2. Mohs Micrographic Surgery: Principles and Technique

Mohs micrographic surgery is typically performed under local anesthesia with the patient fully awake. The visible tumor and surrounding skin are anesthetized through targeted injections. The surgeon then excises the clinically apparent tumor and immediately sends the tissue to an on-site laboratory for histologic processing [3]. Once prepared, the tissue sections are examined microscopically by the Mohs surgeon to identify any residual cancer cells at the peripheral or deep margins. This meticulous analysis allows for the detection of microscopic tumor extensions that may not be visible to the naked eye, including those that are subtle, infiltrative, or located beneath the skin surface. If a residual tumor is identified, additional tissue is removed precisely from the involved margins and re-examined. This iterative process is repeated until all margins are free of the tumor. Because the total number of excision stages required cannot be predicted in advance, patients should anticipate spending several hours, or potentially the entire day, at the dermatology clinic or surgical center [4].

MMS is necessary principally for the treatment of NMSC, such as BCC, but also for all rare skin cancers, just like melanoma (LMM) and dermatofibrosarcoma protuberans and other mesenchymal neoplasies [1]. It is also particularly indicated when skin cancer is located in functionally and aesthetically critical anatomical sites, such as the nose, or in high-risk areas that are cosmetically and functionally sensitive [5]. It is also essential when the tumor involves regions important for daily activities, such as the fingers, or areas that significantly impact appearance.

MMS is also indicated when previous treatments have failed, in the presence of large tumors, or when conventional surgical excision is less likely to achieve complete removal of the neoplasm [2,6].

Although Mohs surgery offers numerous advantages, the choice between this technique and traditional surgery depends on various factors, including the location and size of the tumor, the patient’s general health, and individual preferences [7]. It is essential that patients discuss with their dermatologist to determine the most appropriate treatment approach.

## 3. Appropriate Use Criteria (AUC)

While MMS is widely regarded as the most effective and tissue-sparing treatment for cutaneous malignancies, certain clinical scenarios may warrant the use of less technically demanding and more cost-effective therapeutic alternatives [8]. To address this, the American Academy of Dermatology (AAD), in collaboration with the American College of Mohs Surgery (ACMS), the American Society for Dermatologic Surgery Association (ASDSA), and the American Society for Mohs Surgery (ASMS), developed the Appropriate Use Criteria (AUC) for MMS. These criteria provide evidence-based guidance on the appropriateness of MMS across 270 distinct clinical scenarios, considering factors such as tumor type, location, recurrence status, and patient health profile [9].

The AUC categorizes each scenario into three levels: appropriate, uncertain, and inappropriate. In their 2012 report, the panel deemed 74.1% of scenarios as appropriate, 8.9% as uncertain, and 17.0% as inappropriate. Specifically, for basal cell carcinoma, 53 scenarios were appropriate, 6 were uncertain, and 10 were inappropriate; for squamous cell carcinoma, 102 were appropriate, 7 were uncertain, and 34 were inappropriate; and for lentigo maligna melanoma and melanoma in situ, 10 were appropriate and 2 were uncertain [5].

These criteria aim to optimize the use of MMS by guiding clinicians in selecting patients who are most likely to benefit from this specialized treatment [10]. It is important to note that while the AUC provides valuable decision support, the ultimate choice of treatment should be tailored to the individual patient’s needs and circumstances, incorporating clinical expertise and patient preferences as we show in Table 3 [11].

## 4. Oncologic Efficacy and Comparison with Other Treatments

Mohs micrographic surgery (MMS) is widely regarded as the gold standard for the surgery treatment of cutaneous malignancies, especially for non-melanoma skin cancers (NMSC), owing to its ability to achieve high cure rates to attain optimal oncologic radicality while maximizing tissue preservation and maintaining cost-effectiveness in an outpatient setting [3,4,12].

Mohs micrographic surgery (MMS) offers a clear therapeutic advantage in terms of oncologic efficacy, particularly due to its significantly lower recurrence rates compared to all other treatment modalities for non-melanoma skin cancer (NMSC) [13]. Data from long-term studies indicate that the five-year recurrence rate for primary basal cell carcinomas (BCCs) treated with MMS is approximately 1%, which is markedly lower than those reported for other common approaches. In comparison, standard surgical excision has a recurrence rate of around 10.1%, while electrodessication and curettage, radiation therapy, and cryotherapy have recurrence rates of 7.7%, 8.7%, and 7.5%, respectively [14].

Chren et al. in 2011 [15] studied the recurrence after treatment of non-melanoma skin cancer. A prospective cohort study showed a consecutive sample of all 495 patients with 616 primary NMSCs diagnosed in 1999 and 2000 and treated with electrodessication and curettage (ED&C), excision, or Mohs surgery. Follow-up was available for 608 tumors (99%). Tumor recurrence was determined with validation by clinical examination. The mean age at diagnosis was 71 years; 97% were men. Overall, 127 tumors were treated with ED&C (20.9%); 309 with excision (50.8%); and 172 with Mohs surgery (28.3%). Over the course of the study, 21 tumors recurred (3.5% [95% confidence interval (CI), 2.2–5.2%]): 2 after ED&C (1.6% [95% CI, 0.2–5.6%]), 13 after excision (4.2% [95% CI, 2.2–7.1%]), and 6 after Mohs surgery (3.5% [95% CI, 1.3–7.4%]). Therefore, recurrence of primary NMSC after treatment occurred in less than 5% of tumors. The recurrence rate after ED&C was lower than expected, and the recurrence rate after Mohs surgery was higher than expected. These findings may be related to the risk for recurrence in the treatment groups [15].

Chren et al. [15] also found that curettage as a conservative surgical option, combined with local excision procedures, represents an effective conservative strategy. It offers rapid recovery and minimal systemic impact, and in selected patients demonstrates a favorable risk–benefit profile.

In NMSC, the use of IGRT (Radiation Therapy Technologies) covering CBCT, MRI-Linac, gating, and surface tracking with clinical case series data showed reduced toxicity and preserved survival in prostate cancer, as well as benefits in other tumor localizations [16].

IGRT includes CBCT, MRI-Linac, and surface-guided tracking—significantly improving target precision, allowing for reduced margins and lower toxicity, and enabling daily adaptive treatment planning [16].

This superior efficacy is largely attributed to the precise, margin-controlled technique used in MMS, which allows for real-time histologic assessment of the entire peripheral and deep tumor margins, minimizing the risk of residual cancer cells being left behind [17]. Moreover, evidence suggests that dermatologists, particularly those trained in Mohs surgery, achieve higher rates of complete tumor removal compared to other specialists. For example, previous comparative analyses have shown that dermatologists consistently demonstrate significantly higher complete excision rates for NMSC than otolaryngologists (*p* > 0.02) and plastic surgeons (*p* < 0.0008), further supporting the expertise of dermatologic surgeons in achieving optimal oncologic outcomes [13].

## 5. Training and Accreditation in Mohs Surgery

Properly trained Mohs surgeons are highly specialized in both the pathological and clinical aspects of skin cancer management. Despite its efficacy, MMS presents several technical and procedural challenges that may compromise outcomes if not meticulously addressed [5]. These include potential errors in surgical technique, suboptimal tissue handling, and inaccuracies in histopathological processing—all of which can contribute to tumor recurrence and increased patient morbidity [18]. Achieving optimal results with MMS relies on strict adherence to established surgical protocols, precise histological mapping, and robust quality control throughout the intraoperative process [6,7,19].

In the US and Brazil, only dermatology-trained physicians are allowed to apply for a fellowship in MMS, and a minimum of 12 months of supervised training under the program director is required. The fellow must review all pathology from the surgical cases performed in the training program. Some practical components of the training program are different between the two countries, especially regarding the number of cases (higher in the US) [20].

The main objective of the training is to become able to evaluate the histopathology of the section, especially tumor-free margins (normal skin). Mohs surgeons must also have expertise in skin reconstruction, repairing most of the cases, to optimize functional and aesthetic outcomes [21].

In Brazil, the Brazilian Society of Dermatology offers a certification in micrographic surgery for the dermatologic surgeons who complete the training program [17]. Most programs are affiliated with a residency program. In the US, MMS training programs are accredited by the Accreditation Council for Graduate Medical Education (ACGME) and the American College of Mohs Surgery (ACMS) [22]. While some fellowship programs and private and academic practices were originally accredited by the ACMS, all US-based fellowships now must be ACGME-approved; a few international fellowships still carry an ACMS approval, but not ACGME. Most of these fellowship directors were trained in the US and started fellowships in other countries. Other societies like the American Society for Dermatologic Surgery (ASDS) and the American Society for Mohs Surgery (ASMS) have dermatologic surgical programs but are not formal Mohs fellowships. The ASDS also offers fellowships in cosmetic surgery that may share the same fellowship directors as the ACMS Mohs surgery programs [23,24].

## 6. Prevention of Wrong-Site Surgery

Wrong-site surgery remains a persistent and preventable issue in dermatologic surgery, often arising from patients’ inability to recall biopsy locations. This brief commentary highlights best practices for preoperative site verification, emphasizing the value of photographic documentation and multidisciplinary confirmation to enhance surgical accuracy and reduce medico-legal risk [8,25].

Accurate surgical site identification is a fundamental aspect of preoperative planning in dermatologic procedures. Despite its importance, wrong-site surgery continues to be a notable source of legal claims against dermatologic surgeons. A common contributing factor is the patient’s inability to reliably recall the exact location of a prior biopsy, especially when the lesion has healed or is no longer visible [9,26].

To mitigate this risk, the use of photographs taken at the time of biopsy has become a widely endorsed best practice. These images serve as a precise reference point for surgical planning and ensure continuity of care, particularly when there is a delay between biopsy and excision [27].

In cases where no photographic documentation exists, clinical judgment becomes essential. Mohs surgeons, through experience and visual assessment, can often identify the likely biopsy site [28]. Nonetheless, when doubt remains, confirming the suspected site with the patient—while acknowledging the limitations of patient recall—can serve as an additional safeguard.

Implementing a standardized approach that includes photographic documentation, thorough clinical evaluation, and, when necessary, patient confirmation can significantly reduce the risk of wrong-site surgery and improve overall patient outcomes [29].

## 7. Aesthetic Outcomes and PROMs

MMS is a highly effective and precise technique for the treatment of skin cancer, particularly in cosmetically sensitive areas in the face. While the primary goal of Mohs surgery is the complete excision of the tumor with minimal margins, it is essential to consider the aesthetic outcomes and psychosocial impact of the procedure on patients [30].

Traditional measures of surgical success primarily focus on clinical aspects such as margin clearance and recurrence rates [31]. However, the aesthetic results of surgery and the emotional and psychological effects associated with facial scarring are significant factors influencing overall patient satisfaction and quality of life.

Patient-reported outcome measures (PROMs) are increasingly recognized as valuable tools in assessing outcomes from the patient’s perspective [32]. These instruments provide insights into how patients perceive their appearance and the impact of surgical interventions on their quality of life. In dermatologic and reconstructive surgery, PROMs can address aspects of post-surgical recovery that may not be immediately evident in clinical evaluations [33].

## 8. Validation and Clinical Application of the FACE-Q

One of the most widely utilized PROMs in the context of facial skin cancer surgery is the FACE-Q skin cancer module. This tool was specifically developed to evaluate aesthetic outcomes, including satisfaction with facial appearance, scar quality, and psychosocial distress following facial skin cancer surgery. The FACE-Q allows clinicians to capture data on patient satisfaction with both the functional and cosmetic results of surgery, providing a more comprehensive view of patient outcomes beyond traditional clinical assessments [34].

In addition to the FACE-Q, other PROMs have been used in the evaluation of post-surgical outcomes in Mohs surgery, providing further context for understanding the broader impact of treatment on patients’ lives, and these are [35]:

Skindex-16/Skindex-29: These instruments assess the impact of skin diseases on patients’ quality of life. While originally designed for chronic skin conditions, they have been used to evaluate the psychosocial impact of facial skin cancer and the outcomes of surgical interventions [36].

Dermatology Life Quality Index (DLQI): A widely utilized tool for measuring the effect of skin diseases on daily life, the DLQI is valuable in assessing how skin cancer treatment and scarring affect a patient’s social interactions, work, and overall functioning [37].

SCAR-Q: This tool specifically evaluates scar outcomes, including appearance and symptoms (such as pain and itching), as well as the psychosocial impact of scars on body image [38].

POSAS (Patient and Observer Scar Assessment Scale): This scale includes both patient and clinician assessments of scar quality, covering pain, itching, color, stiffness, and overall appearance. It is particularly valuable for tracking the healing and cosmetic outcomes of post-surgical scars [39].

The FACE-Q is a validated tool for assessing patient-reported outcomes in facial aesthetic surgery. The FACE-Q is a validated and methodologically rigorous patient-reported outcome measure (PROM) specifically developed to evaluate outcomes that are meaningful to individuals undergoing facial aesthetic procedures, both surgical and non-surgical. Its development was grounded in qualitative research, incorporating patient interviews and expert input to ensure content relevance and comprehensiveness [40].

The FACE-Q skin cancer module is a rigorously developed patient-reported outcome (PRO) instrument designed to assess outcomes following facial skin cancer surgery from the patient’s perspective. Developed through concept elicitation interviews with 15 patients and field-tested in a study involving 209 patients, it provides valuable insights into aspects such as appearance satisfaction, quality of life, and patient experience [41].

This modular tool includes five independently functioning scales which are:

(1) Satisfaction with Facial Appearance: Measures overall satisfaction with facial appearance post-surgery.
(2) Appraisal of Scars: Assesses how bothered a patient is by their facial scars.
(3) Cancer Worry: Evaluates concerns about the potential recurrence or severity of skin cancer.
(4) Appearance-related Psychosocial Distress: Captures emotional and social impacts related to facial appearance.
(5) Satisfaction with Information: Assesses satisfaction with information provided about appearance and scar healing.

Additionally, the module includes two checklists:

(1) Sun Protective Behavior: Measures adherence to sun protection practices.
(2) Adverse Effects: Assesses the presence of side effects following skin cancer treatment.

These scales and checklists are designed to be flexible, allowing clinicians and researchers to select the subset most relevant to their specific study or clinical context.

Although Mohs surgery primarily focuses on the removal of skin cancer, the aesthetic outcome is a crucial factor in determining patient satisfaction, especially when the surgery is performed on highly visible areas such as the face. Scarring and changes in appearance can significantly impact patients, leading to emotional challenges related to body image, self-esteem, and social interactions. The integration of patient-reported outcome measures (PROMs) into clinical practice enables a deeper understanding of these psychological and emotional effects, ensuring that patients’ concerns are addressed throughout the entire surgical process and recovery period [42].

Furthermore, PROMs allow healthcare providers to track long-term effects of the surgery, including scar formation, psychosocial distress, and overall satisfaction. This patient-centered approach not only enhances the quality of care but also provides valuable data that can guide refinements in surgical techniques and post-operative care, ultimately optimizing both functional and aesthetic outcomes for patients.

The instrument comprises a series of independently functioning scales that assess multiple dimensions of the patient experience. These include satisfaction with facial appearance, health-related quality of life, psychological well-being, recovery, adverse effects, and satisfaction with the care process. Each domain is further subdivided into targeted scales that evaluate specific facial regions and functions, thereby allowing for a nuanced and patient-centered assessment of treatment outcomes [19,43].

The FACE-Q has undergone extensive psychometric evaluation to establish its reliability, validity, and responsiveness, and has been widely adopted in clinical research and practice. It enables clinicians and researchers to systematically capture the impact of aesthetic interventions on patient satisfaction and quality of life [20].

In recognition of its scientific rigor and utility, the United States Food and Drug Administration (FDA) has qualified several FACE-Q scales as Medical Device Development Tools (MDDTs), affirming their relevance in the regulatory evaluation of medical devices and contributing to the standardization of outcome reporting in aesthetic medicine [21,44].

## 9. Comparative Aesthetic Outcomes: MMS vs. Standard Excision

As demonstrated by Alam, M. et al. [20], FACE-Q questionnaires could be used for the assessment of patients’ satisfaction with their facial appearance in general and in social situations. They reported that when overall patient satisfaction with facial appearance was assessed, no statistically significant differences were observed between baseline and the two post-operative follow-up time points. Rasch-transformed scores were 70.27 (SD 17.98) at baseline, 68.53 (SD 17.34) at one month post-surgery, and 73.05 (SD 22.32) at three months post-surgery [22,45].

The primary finding of this study was that patients reported significantly greater satisfaction with the aesthetic outcome of facial skin cancer surgery—whether performed via Mohs micrographic surgery (MMS) or standard excision (SE)—at three months post-surgery compared to the one-month follow-up [23,24].

The observed increase in patient satisfaction with their facial appearance over the two months following surgery may be explained by the hypothesis that scars are more erythematous and noticeable within the first three months post-surgery, as noted in a study by Lewis et al. [23]. Additionally, collagen production peaks around 21 days after the skin injury, which results in a more pronounced, thickened appearance of scars. Following this period, collagen synthesis begins to slow down, and the density of capillary redness decreases as the inflammatory phase regresses. During this time, scars transition into the maturation phase, becoming less visible [26,46].

Furthermore, the results indicate that patients’ satisfaction with the aesthetic outcomes improves significantly in cases of larger facial defects (>10 mm) between the one- and three-month follow-up periods.

The study also demonstrated that there were no statistically significant differences in patient satisfaction with the aesthetic outcome between Mohs micrographic surgery (MMS) and standard excision (SE) at either post-operative follow-up. However, it is important to note that this study was not specifically designed to compare MMS and SE in terms of aesthetic results. Given that MMS is not yet widely adopted as a standard treatment modality in many European hospitals, the findings of this study may be particularly relevant in clinical settings where SE remains the primary therapeutic option [28,47].

In the context of facial skin cancer surgery, the aesthetic outcome plays a pivotal role in overall patient satisfaction. While Mohs micrographic surgery (MMS) is renowned for its high cure rates and tissue-conserving technique, its cosmetic benefits compared to traditional surgical excision (TSE) have been the subject of increasing clinical interest [29,48]

Recent prospective studies have explored the psychosocial and aesthetic impact of these procedures, particularly over extended follow-up periods. In evaluations that included domains such as quality of life, body image, perceived stigma, and satisfaction with facial appearance, findings revealed no significant differences between patients treated with MMS and those undergoing TSE. Both groups reported progressive improvements over time, suggesting that the choice of surgical technique does not significantly affect long-term psychosocial outcomes [30].

Moreover, assessments using the FACE-Q, a validated patient-reported outcome instrument specific to facial aesthetic surgery, demonstrated comparable levels of satisfaction among patients treated with either MMS or TSE [30,49] This reinforces the notion that both approaches yield similar results in terms of patient-perceived cosmetic outcomes.

From a reconstructive standpoint, MMS has the advantage of producing smaller surgical defects due to its precise, stepwise excision of cancerous tissue. This can facilitate more conservative closures and potentially optimize aesthetic results. However, comparative studies have shown that this technical benefit does not necessarily translate into significantly superior aesthetic outcomes when compared to conventional excision—particularly when both are performed with attention to cosmetic detail [32,50].

Taken together, the evidence suggests that while MMS offers important oncological advantages, including margin control and tissue preservation, the aesthetic results achieved are generally comparable to those obtained with traditional excision, provided that both procedures are executed by skilled surgeons employing appropriate reconstructive strategies [11,31].

Additionally, no significant differences in patient-reported satisfaction were observed across different facial zones. Nevertheless, to more accurately identify the facial aesthetic subunits most affected by skin cancer surgery, studies with larger sample sizes are warranted.

Although most patients reported a return to baseline levels of satisfaction with their general facial appearance and appearance in social situations by 3 months post-operatively, a longer follow-up period would be necessary to fully evaluate the long-term aesthetic impact of facial NMSC surgery [32,50].

An extensive analysis of the existing literature comparing Mohs micrographic surgery (MMS) to traditional surgical excision confirms the clinical superiority of MMS in achieving both the highest initial cure rates and lowest recurrence rates for non-melanoma skin cancers (NMSC). The margin-controlled, layer-by-layer approach inherent to MMS enables complete evaluation of 100% of surgical margins during the procedure, reducing the likelihood of residual tumor tissue and thereby minimizing the need for further intervention. This level of precision is particularly advantageous in cosmetically and functionally sensitive areas, such as the face, where tissue preservation and recurrence prevention are essential.

## 10. Economic Considerations

From an economic perspective, the assumption that MMS is more costly than standard excision is increasingly being challenged. Although the per-procedure cost of MMS may be perceived as higher, especially in outpatient settings, studies have shown that MMS is often more cost-effective over the long term. Traditional surgical excision frequently incurs additional costs, including ambulatory surgical center (ASC) facility fees, pathology charges, and the potential necessity for re-excision if tumor margins are not clear. In contrast, the immediate and complete margin assessment provided by MMS typically avoids these follow-up procedures, thus lowering the overall cost of care [11].

MMS represents a model of such efficiency: it offers high cure rates, low recurrence, minimized tissue sacrifice, and often a single-treatment resolution—all of which contribute to greater patient satisfaction and reduced long-term expenditure. It is important to underline that MMS is often more cost-effective over the long term [51].

In a comprehensive review of the literature, Mohs micrographic surgery (MMS) was found to be cost-equivalent to excision with permanent sections, approximately 12% less expensive than office-based excision with frozen sections, and about 27% less costly than excision with frozen sections performed in an ambulatory surgical center (ASC). Additionally, 32% to 39% of cases treated with surgical excision required a subsequent procedure to achieve clear margins. These follow-up procedures often involved the removal of a greater volume of tissue, with potential implications for functional preservation and cosmetic outcomes, which are challenging to quantify. This analysis underscores the cost-effectiveness of MMS, highlighting its value not only in achieving high initial cure rates and low recurrence rates but also in reducing the need for additional surgical interventions. Such efficiencies are particularly pertinent in the current healthcare climate, where financial considerations and declining reimbursement rates may impact the delivery of optimal care for non-melanoma skin cancers [52].

## 11. Conclusions

Mohs micrographic surgery is a highly effective treatment modality for a wide range of cutaneous neoplasms. It has demonstrated statistically significant superiority over standard surgical excision, particularly in the management of recurrent and high-risk non-melanoma skin cancers (NMSC).

Preventing wrong-site surgery begins with precise and proactive preoperative planning. Integrating photographic documentation into routine biopsy workflows, combined with clinical expertise and patient communication, forms a reliable framework for ensuring surgical accuracy and patient safety [33,53].

Evaluating outcomes after Mohs surgery requires more than traditional clinical metrics such as tumor clearance and recurrence rates. To fully understand the impact of surgery—particularly in cosmetically sensitive areas like the face—it is essential to incorporate Patient-Reported Outcome Measures (PROMs) [34]. Tools such as the FACE-Q, DLQI, and POSAS provide critical insights into patients’ perceptions of their aesthetic results and emotional well-being. By including these measures in routine clinical practice, healthcare providers can ensure a more holistic, patient-centered approach to care [35,54].

Ultimately, the integration of PROMs leads to a deeper understanding of patient experiences, helping to enhance satisfaction, support long-term recovery, and improve overall quality of life following facial skin cancer surgery [36].

While Mohs micrographic surgery (MMS) offers distinct oncological advantages, particularly in margin control and tissue preservation, the aesthetic results it provides are, in most cases, comparable to those achieved through traditional excision (TSE). This holds true as long as both surgical techniques are performed by experienced surgeons who employ appropriate reconstructive strategies. Therefore, MMS should be considered not only for its superior tumor control but also for its comparable aesthetic outcomes, ensuring that patient satisfaction is optimized across both functional and cosmetic dimensions.

No significant differences in patient satisfaction with aesthetic outcomes were observed across different facial zones. However, to more precisely identify the facial areas—or more specifically, the aesthetic subunits—most affected by skin cancer surgery in terms of perceived aesthetic impact, studies involving larger patient populations are needed. Moreover, while most patients report a return to baseline satisfaction with their facial appearance, both generally and in social contexts, within three months post-surgery, a longer follow-up period would be preferable to more thoroughly assess the long-term aesthetic effects of facial non-melanoma skin cancer (NMSC) surgery [55].

In conclusion, while Mohs micrographic surgery (MMS) offers clear oncologic advantages such as superior margin control and tissue preservation, its aesthetic outcomes are generally comparable to those of traditional surgical excision when both are performed by experienced surgeons using appropriate reconstruction techniques. To further clarify the relationship between tumor characteristics—particularly lesion size—and patient satisfaction with postoperative outcomes, future research should involve larger study populations and employ standardized clinical instructions to minimize variability [56,57].

Future advancements are expected to include increased integration of noninvasive imaging techniques, immunohistochemical staining, and digital technologies to further enhance diagnostic precision and surgical outcomes.

## Figures and Tables

**Table 1:** Summary of Study Selection Process.

Stage	Number of Studies
Records identified	150
Duplicates removed	20
Screened for eligibility	130
Studies included	45

**Table 2:** Summary of Key Findings.

Outcome	MMS	Standard Excision
Recurrence Rates	Lower	Higher
Long-Term Cost-Effectiveness	More cost-effective	Less cost-effective
Aesthetic Outcomes	Comparable	Comparable

**Table 3:** Considerations of MMS in NMSC. Comparative Table: MMS vs. Standard Surgical Excision in NMSC Treatment.

Aspect	Mohs Micrographic Surgery (MMS)	Standard Surgical Excision (SE)
Oncologic Efficacy	10-year recurrence rates: 4.4% for primary BCC, 3.9% for recurrent BCC	10-year recurrence rates: 12.2% for primary BCC, 13.5% for recurrent BCC
Quality of Life (QoL)	QoL improvement similar to SE post-treatment	QoL improvement similar to MMS post-treatment
Cost Considerations	Higher initial costs due to longer procedure time and pathology	Lower initial costs, but may incur additional expenses for re-excisions and complications
Aesthetic Outcomes	Smaller surgical defects, better cosmetic results	Larger surgical defects, potentially more noticeable scarring
Indications	Recommended for high-risk, recurrent, or facial BCCs	Suitable for low-risk, well-defined BCCs
Guideline Recommendations	Preferred for high-risk and recurrent BCCs	Suitable for low-risk BCCs

## Data Availability

Not applicable.
